# Utilization of Mammography, Sonography and Radiology Services before and after Health Sector Evolution Plan in Iran

**Published:** 2018-03

**Authors:** Ahmad KALATEH SADATI, Shadi HAJIZAMANI, Reza SADEGHIMEHR, Saeid AGHAJANIAN, Mahbobeh AMOZAGAR

**Affiliations:** 1. Dept. of Sociology, Faculty of Humanities & Social Sciences, Yazd University, Yazd, Iran; 2. Health Policy Research Center, Institute of Health, Shiraz University of Medical Sciences, Shiraz, Iran

## Dear Editor-in-Chief

Increasing for inpatient utilization after Health Sector Evolution Plan in Iran was done in 2014. “It seems that inpatient health coverage by government increase the possibility of induced demand by service provider after Iranian health care reform are important reasons for this changes in utilization index” (1). Since the health costs in countries is an important factor that drags the individuals and households below the poverty line (2), one goal of Universal Health Coverage (UHC) of WHO is how to provide good health services with less expensive costs. In fact, UHC is an attempt to achieve social justice by facilitating access to health services (3) by accessibility and poverty prevention because of costs.

On the other hand, the statistics in Iran showed that individuals are dragged to the poverty line annually because of these costs (4). Health sector Evolution Plan (HSEP) was designed in 2014 for decreasing the problem. The most important strategy of the plan was the resources transferring to public hospitals and enable them in order to give good services at different levels by decreasing to 10% of the costs. This leads to that the patients turn over to the public hospitals increased and it caused health service utilization increment. The main question of this contribution is evaluation of plan based on UHC’s view with UI of mammography, sonography, and radiology during the 6 months before and after reform.

Data for this descriptive study was gathered from R&D department of Shiraz University of Medical Sciences (SUMS) and private hospitals. Data were analyzed with evaluation utilization index (UI) formula is the number of services 6 months after reform is divided by the number of services 6 months before reform in the same public or privet hospitals.

The private inpatient utilization in radiology increased from 48% to 57% after the plan. In addition, we observe an increment in private inpatient for sonography services from 19% to 25% which means a decrement in utilization pattern in public. However, we observe a 1% increment in public hospitals mammography (Table 1).

**Table 1:** UI of mammography, sonography, and radiology

***Type of service***	***UI inpatient governmental hospitals***	***UI outpatient governmental hospitals***	***UI inpatient private hospitals***	***UI outpatient private hospitals***
Mammography	1.51	1.38	1.07	-
Sonography	0.83	1.03	3.45	1.23
Radiology	0.93	0.94	1.18	1.06

There is an increment in public inpatients & out-patient’s mammography services while there is no increment in radiology and sonography. The increment relevant meet the needs better after the plan, while we see no increment in sonography and radiology services. We have had an increment in private hospitals. The utilization of the privet hospital increased despite the plan does not include private hospital. The utilization and induced demand are not increased for public hospital and in our opinion; it is unfair for two reasons: 1. the descending tax for financing in this plan; 2. the health resource spending somewhere that is not a priority.

In addition, this does not include special group and this service does not result in catastrophic household expenditure.

If the UI is not increased, the induce–demand possibility is rejected and it is unfair. There is no need to be under the cover of these services. In addition, those services are not settled into catastrophic expenditure category. Generally, the patients out of pocket (OOP) are more than 10% because there is no plan for privet and outpatient in the plan policies.

Based on UHC’s view can say that HSEP is not in justice services. The decrement of the services terrifies by the public hospitals. All of the people with different social and economic classes come to the public hospitals for service delivery. It will be unfair to get the utility of services if the decision-making is without the consideration of the service distribution injustice. An important question is that whether the health source distribution should be accursed in all health services and cases or just in special cases. A more important question is that whether sonography and radiology are the reasons for expensive costs and do they drag the households under the poverty line. On the other hand, the reform led to induce demand directly. Transferring the sources to the public hospitals, the number of the patients and the doctors to use the sources and services increased which increased resource wastage generally we can say that health reform plan due to a general pattern for all inpatient and health services and also lack of comprehensive for strategic purchasing the plan cannot support vulnerable people against catastrophic expenditure. In addition, the reform increased level of inequality in society which leads to formation of class society (5). We suggest to the politicians in order to improve the justice. In addition, it helps us not to omit the expensive cost services in the future. In order to administer the justice in UHC, it is a good idea to have more intention to the strategic purchasing with a suitable price and service for a suitable group and provide good service packages with the maximum effectiveness. Therefore, policy-makers must promote and make better decision in health reform.
